# Compensatory and Dynamic Cerebellar Responses to Striatal Lesions in Experimental Parkinsonism

**DOI:** 10.1007/s12311-026-01971-x

**Published:** 2026-03-21

**Authors:** Luis I. García, Gerardo Marín, Cristofer Zárate-Calderón, Iraís Viveros-Martínez, Mario E. Valerio-Nolasco, Luis Beltrán-Parrazal, Donaji Chi-Castañeda

**Affiliations:** 1 Instituto de Investigaciones Cerebrales, Universidad Veracruzana, Xalapa, Veracruz 91190 México; 2 Departamento de Neurocirugía, “Hospital Regional 1◦ de Octubre”, Instituto de Seguridad y Servicios Sociales de los Trabajadores del Estado (ISSSTE), 07300 México City, México

**Keywords:** Cerebellum, Cerebellar nuclei, Inferior olive, Parkinsonian disorders, Tremor, Electrophysiology, Compensatory effect

## Abstract

Parkinsonian symptoms such as tremors, rigidity, bradykinesia, and postural instability typically arise from basal ganglia dysfunction, but growing evidence suggests cerebellar circuits also play a key role. Here, we investigated multiunit activity (MUA) in the inferior olive (IO), dentate nucleus (DN), and Crus II of the cerebellum in a rat model of tract lesion-induced parkinsonism triggered by ventrolateral striatum (VLS) injury. Thirty-six male Wistar rats were divided into a Lesion group and an Intact group. Monopolar electrodes were implanted to record MUA in IO, DN, or Crus II for four consecutive weeks. Basal and tremor-associated signals were analyzed using generalized linear models and post hoc comparisons. In rats with VLS lesions, IO activity initially increased and then declined over time, whereas DN activity remained consistently elevated, suggesting compensatory upregulation. Crus II showed no significant shifts in baseline activity. During tremor episodes, all three structures exhibited distinct temporal fluctuations in MUA. These findings reveal cerebellar structure-specific responses to striatal injury and highlight the cerebellum’s role in both the acute and chronic phases of Parkinsonian motor dysfunction. Careful consideration of possible inflammatory responses to electrode implantation remains essential for future studies.

## Introduction

Parkinsonism is a clinical syndrome observed across various neurological conditions, most prominently in Parkinson’s disease (PD). Nevertheless, it also manifests in secondary forms such as drug-induced parkinsonism, vascular parkinsonism, traumatic brain injury-induced parkinsonism, and atypical Parkinsonian syndromes [1–3] . This syndrome is commonly associated with basal ganglia (BG) dysfunction and is characterized by tremor, muscular rigidity, postural instability, and bradykinesia [4].

In rat models, tremulous jaw movements (TJMs), which are mandibular movements resembling mastication but lacking a functional purpose, are characteristic behavioral markers used to study resting tremor [5]. These tremors can be induced pharmacologically by manipulating striatal dopaminergic systems [6] , employing neurotoxins such as 6-hydroxydopamine or 1-methyl-4-phenyl-1,2,3,6-tetrahydropyridine (MPTP), or by electrolytic and mechanical lesions specifically targeting the ventrolateral striatum (VLS) [5, 7–10] –. The tremors induced by these standardized animal models display behavioral characteristics comparable to those observed in resting tremor [8–10] . However, current perspectives on Parkinsonian pathophysiology have shifted towards a broader network dysfunction, placing the cerebellum as a critical protagonist alongside the BG [5, 8, 11] . Rather than operating in isolation, these structures maintain continuous, bidirectional interactions mediated by specific synaptic pathways, such as projections from the dentate nucleus (DN) to the striatum and from the subthalamic nucleus (STN) to the cerebellar cortex [12, 13] . While this integrated network undoubtedly supports cognitive and sensory domains [14–18] , its role in motor control is paramount, particularly regarding how cerebellar circuits might reorganize to compensate for striatal deficiency.

Building upon evidence that VLS manipulation triggers immediate cerebellar activation [5] , the present study analyzed the Crus II lobule, which, despite not being directly associated with motor functions, has been established as part of the anatomical communication circuit between the cerebellum and the striatum [12] . In addition, the inferior olive was evaluated due to its essential role as the primary source of climbing fibers [19] , along with the dentate nucleus, given its anatomical relevance and its proposed involvement in tremor generation [9, 13] , 

.

## Materials and Methods

### Experimental Subjects

This research was approved by the Institutional Animal Care and Use Committee (CICUAL-CICE) of the Universidad Veracruzana under approval number 2018-003. Thirty-six male Wistar rats (initial weight: 250–350 g) were used, housed individually in acrylic cages (44 × 33 × 20 cm) with sawdust bedding (Rismart, Mexico City, Mexico). The animals were maintained under an inverted light-dark cycle (12 h light/12 h dark) and had free access to commercial rodent chow (Purina Rodent Chow^®^) and purified water. Animal care and handling were carried out in strict compliance with the Mexican Official Standard NOM-062-ZOO-1999 and the Guide for the Care and Use of Laboratory Animals. All animals remained in the animal facility of the Brain Research Institute of the Universidad Veracruzana.

### Study Groups

The rats were randomly assigned to two groups: Intact (*n* = 18) and Lesion (*n* = 18).

In the Lesion group, subjects received a bilateral tract lesion, defined as the linear tissue disruption generated by the penetrating descent of an electrode. The passage of this instrument causes damage along the entire trajectory toward the ventrolateral striatum (VLS). Specifically, an electrode (FHC, Inc., USA; diameter: 250 μm; impedance: 3 MΩ) was lowered into the VLS (Table 1) and maintained in position for 15 s before withdrawal, thereby producing a tract lesion along its path. In addition to the lesion, recording electrodes were implanted in one of the three cerebellar structures of interest, along with their corresponding reference electrodes.

**Table 1:** Coordinates of the structures

Structure	AP [mm]	ML [mm]	DV [mm]
VLS	-0.48	± 4.40	-6.80
Crus II	-14.00	3.40	-5.00
OI	-11.80	0.80	-11.00
DN	-11.30	3.40	-6.40

This table presents the coordinates used in the present study, referenced from the Bregma point. The values were obtained from The Rat Brain in Stereotaxic Coordinates. Abbreviations: *VLS*, Ventrolateral Striatum; *IO*, Inferior Olive; *DN*, Dentate Nucleus; *AP*, Anteroposterior; *ML*, Mediolateral; *DV*, Dorsoventral [20].

In the Intact group, subjects did not receive a striatal lesion. Importantly, animals in this group underwent the same surgical procedures for cerebellar electrode implantation as those in the Lesion group, including anesthesia and stereotaxic fixation. Thus, they served as controls for surgical stress and the presence of chronic implants, allowing the isolation of effects specifically attributable to the VLS lesion.

Each group was further divided into three subgroups based on the cerebellar area to be analyzed (IO, DN, and Crus II), with each subgroup comprising six animals per structure (*n* = 6). A recording electrode and a corresponding reference electrode were implanted in every rat across all groups.

### Electrode Development

For MUA recording, stainless steel monopolar electrodes were fabricated (FHC, Inc., USA; diameter: 250 μm; impedance: 3 MΩ). These electrodes were insulated with epoxy resin, leaving an exposed conductive tip of approximately 1 mm for neuronal contact. The length of each electrode was specifically determined to reach the dorsoventral coordinates of the target brain structure. One end of each electrode was connected to a male copper connector. The conductivity of each electrode was verified before implantation. The reference electrode consisted of a 2 mm long stainless-steel screw with a wire wrapped around its head and connected to a male connector.

### Electrode Implant and Recording

#### Electrode Implant for MUA

The stereotaxic neurosurgical technique employed here follows the protocol outlined by Vásquez-Celaya [5] . Electrodes were implanted unilaterally into the right hemisphere, aiming at Crus II, the IO, and the DN. Both Intact and Lesion animals underwent this implantation; however, in the Lesion group, a bilateral craniotomy was first performed. Following that, electrodes were advanced into the VLS without applying electrical stimulation, thereby producing a tract lesion without requiring electrode anchoring. The specific stereotaxic coordinates used for the VLS, Crus II, IO, and DN are detailed in Table 1.

#### MUA Recording

The MUA recordings in the IO, Crus II, and DN began 72 h after surgery and were conducted once weekly for four consecutive weeks. Before each recording session, the rats were placed in an acrylic box (30 × 30 × 30 cm) for five minutes to acclimate. After this period, the implanted electrodes were connected to an amplifier (15A54, 15LT Grass Technologies, Inc., West Warwick, RI, USA) and an audio monitor (AM9 Grass Technologies, Inc., West Warwick, RI, USA). The amplified signal was digitized and displayed on a computer (HP 6730b, Palo Alto, CA, USA) using a Polyview PVA16 adapter system (Grass Technologies, Inc., West Warwick, RI, USA). The identification of TJMs episodes was based on standardized observations by two expert evaluators who were blinded to the experimental condition. Detection criteria were based on the specific frequency and amplitude patterns previously validated in this model by Herrera-Meza et al., where VLS lesions were shown to induce tremors in the 3–9 Hz range [8] . Only episodes that matched these phenotypic characteristics were selected for electrophysiological correlation.

### Histological Verification

Animals were euthanized by overdose of sodium pentobarbital (170 mg/kg, i.p.; Cheminova, Mexico) and transcardially perfused with 0.9% saline solution followed by 4% paraformaldehyde. Brains were subsequently removed and post-fixed for histological processing.

To verify the extent of the tract lesion in the VLS and the correct placement of cerebellar electrodes, coronal Sect. (45 μm thick) were obtained using a cryostat (Leica^®^ CM1850, Nussloch, Germany) at − 24 °C. Sections were mounted on glass slides and stained using a modified Nissl protocol.

The staining procedure consisted of: (1) washing in distilled water for 1 min; (2) immersion in (2) immersion in 0.5% cresyl violet acetate solution (Sigma-Aldrich, St. Louis, MO, USA; catalog no. C5042-10G) for 8 min; (3) dehydration in 70% ethanol for 15 s; and (4) dehydration in 100% ethanol for 10 s. Sections were then cleared in xylene and coverslipped with Permount mounting medium.

Tissue was examined under a light microscope (AX70, Olympus Co., Shinjuku, Tokyo, Japan) to confirm electrode placement and to quantify the lesion area.

### Statistical Analysis

For the analysis of multiunit activity data, ten representative traces from each week for each animal, corresponding to the behaviors of interest, were randomly selected. The dependent variable (maximum amplitude) was analyzed using a Generalized Linear Model (GLM) with fixed and nested factors, implementing a hierarchical design [5, 17] . Separate analyses were performed for basal activity and for episodes of TJMs.

The model for basal activity was specified as:

$$y=G+W+G\times W+R\left[W\right]+T\left[R\right]+\in$$

For TJMs, the model was:

$$y=W+R\left[W\right]+T\left[R\right]+\in$$

Where:

$y$: maximum amplitude of the MUA

$G$: fixed factor representing the experimental group (Intact, Lesion)

$W$: fixed factor representing recording week

$G\times W$: interaction between group and week

$R\left[W\right]$: random factor for rat nested within week

$T\left[R\right]$: random factor for trace nested within rat

$$\in$$: residual error

Normality assumptions were checked using the Lilliefors test; if the data did not meet these assumptions, a rank transformation was applied prior to model use. A Tukey *post hoc* test was then applied to identify significant differences between groups and weeks, using $\alpha<0.05.$ Analyses were conducted separately for each brain structure (IO, Crus II, DN). All statistical analyses were performed with JMP 14 (SAS Inc., Cary, NC, USA, 2019).

## Results

For each behavior, the corresponding analysis was performed by structure, focusing primarily on the implications of the *Week* × *Group* interaction effect for basal activity and the effect of *Week* for TJMs activity.

### Basal Activity

**Fig. 1:**
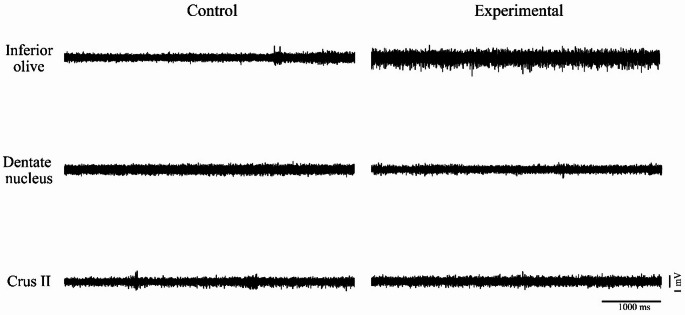
Representative multi-unit activity (MUA) traces recorded during periods of complete rest in the inferior olive, dentate nucleus, and Crus II. The horizontal calibration bar corresponds to 1000 ms and the vertical calibration bar to 1 mV

For basal activity, 1440 characteristic traces were analyzed (480 per structure), with a focus on MUA amplitude (Fig. 1). The results obtained for each structure were as follows:

#### Inferior Olive

**Fig. 2:**
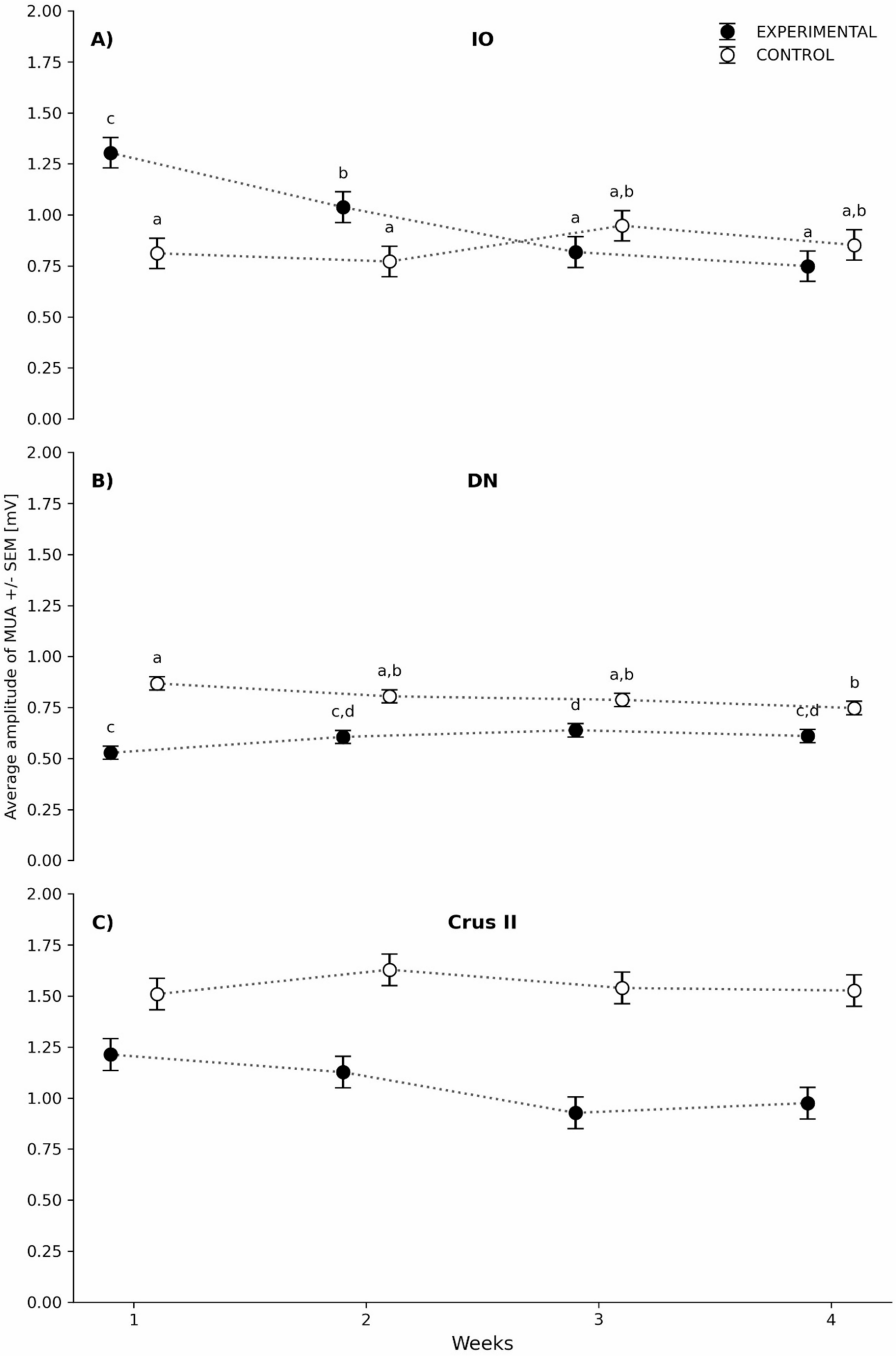
Maximum basal MUA amplitude (± SE) recorded across the four weeks of the study in: **A**) inferior olive (IO), **B**) dentate nucleus (DN), and **C**) Crus II. White circles represent the control group, and black circles represent the experimental group. The X-axis shows the recording weeks, and the Y-axis indicates MUA amplitude (mV). Different letters denote significant differences. Statistical comparisons were performed between groups within each structure, with a significance level of *p* < 0.05

For basal activity in the IO, the analysis revealed a statistically significant interaction between *Group* and *Week*, indicating that the effect of the tract lesion varied over time (Table 2). Significant main effects were also found for both *Group* and *Week*. The Tukey *post hoc* test showed that the maximum amplitude of the MUA in the IO for Weeks 1 (W1) and 2 (W2) was significantly higher in the Lesion group compared to those same weeks in the Intact group. However, no significant differences were observed in Weeks 3 (W3) and 4 (W4). Regarding intragroup comparisons in the Lesion group, there was a progressive decrease in MUA over time: W2 was lower than W1, W3 was lower than W2, and W4 showed stabilization when compared to W3 (Fig. 2A).

**Table 2:** Results of the statistical analysis of effects on basal activity across cerebellar structures

Structure	Source	DF	Ratio F	*p*-value
IO	GROUP	1	4.5206	0.0341*
	WEEK	3	3.4056	0.0177*
	GROUP*WEEK	3	10.2230	< 0.0001*
	RAT[WEEK]	20	14.4657	< 0.0001*
	TRACE[RAT]&Random	54	0.5293	0.9975
DN	GROUP	1	94.2013	< 0.0001*
	WEEK	3	1.6974	0.1670
	GROUP*WEEK	3	6.3969	0.0003*
	RAT[WEEK]	20	13.4739	< 0.0001*
	TRACE[RAT]&Random	54	0.3239	1.0000
Crus II	GROUP	1	141.7359	< 0.0001*
	WEEK	3	0.3507	0.7887
	GROUP*WEEK	3	0.5187	0.6696
	RAT[WEEK]	24	22.5169	< 0.0001*
	TRACE[RAT]&Random	63	0.4432	0.9999

Summary of GLM effects for *Group*,* Week*, and their interaction, including nested factors

#### Dentate Nucleus

For basal activity in the DN, the analysis revealed a statistically significant interaction between *Group* and *Week* (Table 2), indicating that the effect of the tract lesion on MUA amplitude varied over time. A strong main effect of *Group* was also observed, whereas no significant effect of *Week* was detected as a main factor. According to the Tukey *post hoc* analysis, the maximum MUA amplitude was lower in the Lesion group than in the Intact group across all four weeks. Conversely, in the intragroup comparison within the Lesion group, there was a relative increase from W1 to W2 and then a stabilization from W2 to W3, with no significant differences observed (Fig. 2B).

#### Crus II

For basal activity in Crus II, the analysis showed no significant interaction between group and week, and no main effect of week (Table 2), indicating that the temporal evolution of MUA amplitude was not significantly different between groups. However, a strong main effect of group was found, suggesting an overall difference in MUA amplitude between Intact and Lesion animals. Due to the absence of a significant group-by-week interaction, no *post hoc* comparisons were performed (Fig. 2C).

In the analysis of nested factors for basal activity, the interaction between rat and week (*Rat[Week]*) was highly significant in all examined structures (IO, DN, and Crus II), as detailed in Table 2. In contrast, the trace-within-rat factor (*Trace[Rat]*) did not show statistically significant differences in any structure analyzed.

### Mandibular Tremor

**Fig. 3:**
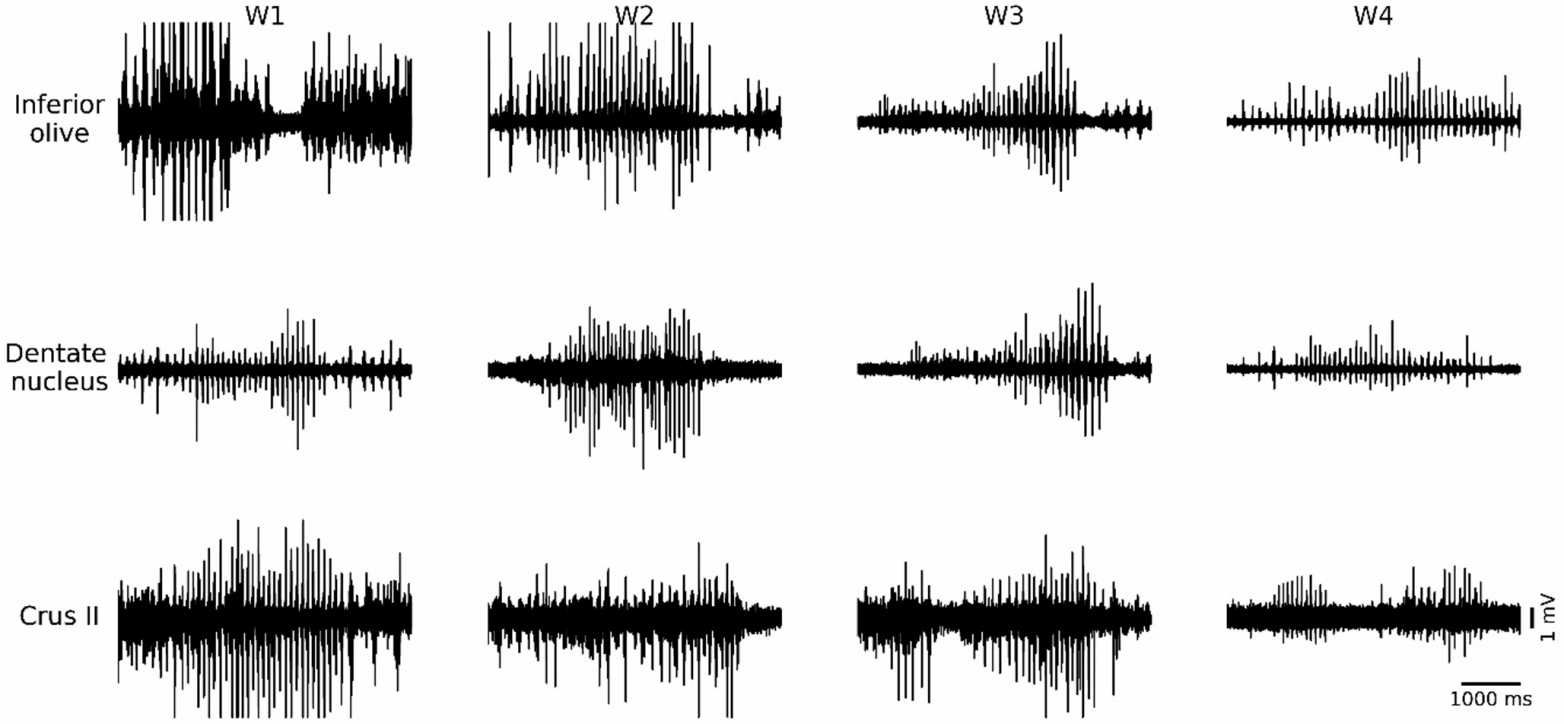
Representative MUA traces recorded during episodes of mandibular tremor in the inferior olive, dentate nucleus, and Crus II across the four weeks of the study. W1, W2, W3, and W4 correspond to weeks 1, 2, 3, and 4, respectively. Horizontal calibration bar: 1000 ms; vertical calibration bar: 1 mV

For TJMs, 720 characteristic tremor traces were analyzed in the Lesion group focusing specifically on the maximum MUA amplitude (Fig. 3). The statistical analysis revealed significant effects of *Week* in all three structures (Table 3). Specifically, in the IO, DN, and Crus II, *Week* had a highly significant effect, and *Rat-within-Week* variability was likewise significant. In contrast, *Trace-within-Rat* variance was non-significant in all structures.

**Table 3:** Results of the statistical analysis of effects on TJMs across cerebellar structures

Structure	Source	DF	Ratio F	*p*-value
IO	WEEK	3	30.5010	< 0.0001*
	RAT[WEEK]	20	14.1454	< 0.0001*
	TRACE[RAT]&Random	54	0.9493	0.5775
DN	WEEK	3	22.9207	< 0.0001*
	RAT[WEEK]	20	11.3738	< 0.0001*
	TRACE[RAT]&Random	54	0.9278	0.6168
Crus II	WEEK	3	33.9125	< 0.0001*
	RAT[WEEK]	20	15.6440	< 0.0001*
	TRACE[RAT]&Random	54	1.2354	0.1582

Summary of GLM effects for *Week* and nested factors

**Fig. 4:**
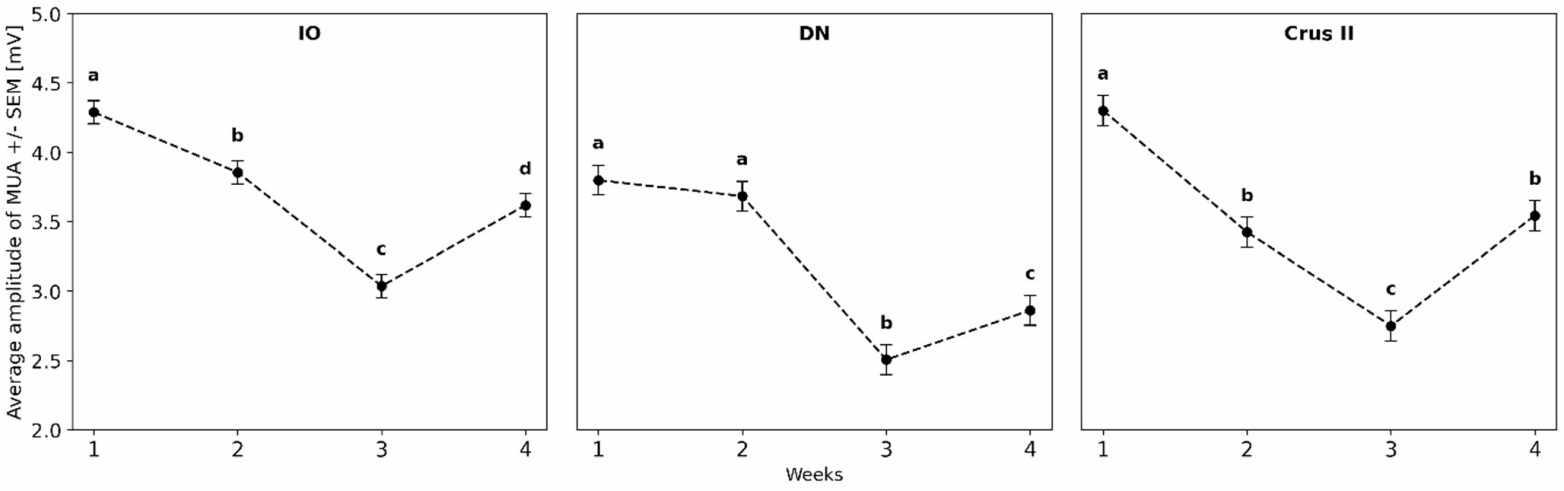
MUA amplitude (mV) ± SE recorded during mandibular tremor expression in the inferior olive (IO), dentate nucleus (DN), and Crus II across four weeks. The X-axis represents the recording weeks, and the Y-axis shows MUA amplitude (mV). Different letters denote significant differences (*p* < 0.05). Statistical comparisons were performed across weeks for each structure independently

The *post hoc* Tukey tests revealed structure-specific temporal dynamics: in the IO and Crus II, the mean maximum amplitude of the MUA in W2 was significantly lower than in W1, while in the DN it remained unchanged during that period. In W3, all structures exhibited a decrease compared to W2. Finally, in W4, all structures demonstrated a significant increase in amplitude relative to W3 (Fig. 4).

Importantly, tremor-related activity was restricted to the Lesion group. Intact animals did not exhibit mandibular tremors or jaw chattering comparable to those observed in lesioned rats, and no tremor-like patterns were detected in their electrophysiological recordings. Therefore, tremor-related analyses were focused exclusively on the Lesion group.

### Histological Verification

**Fig. 5:**
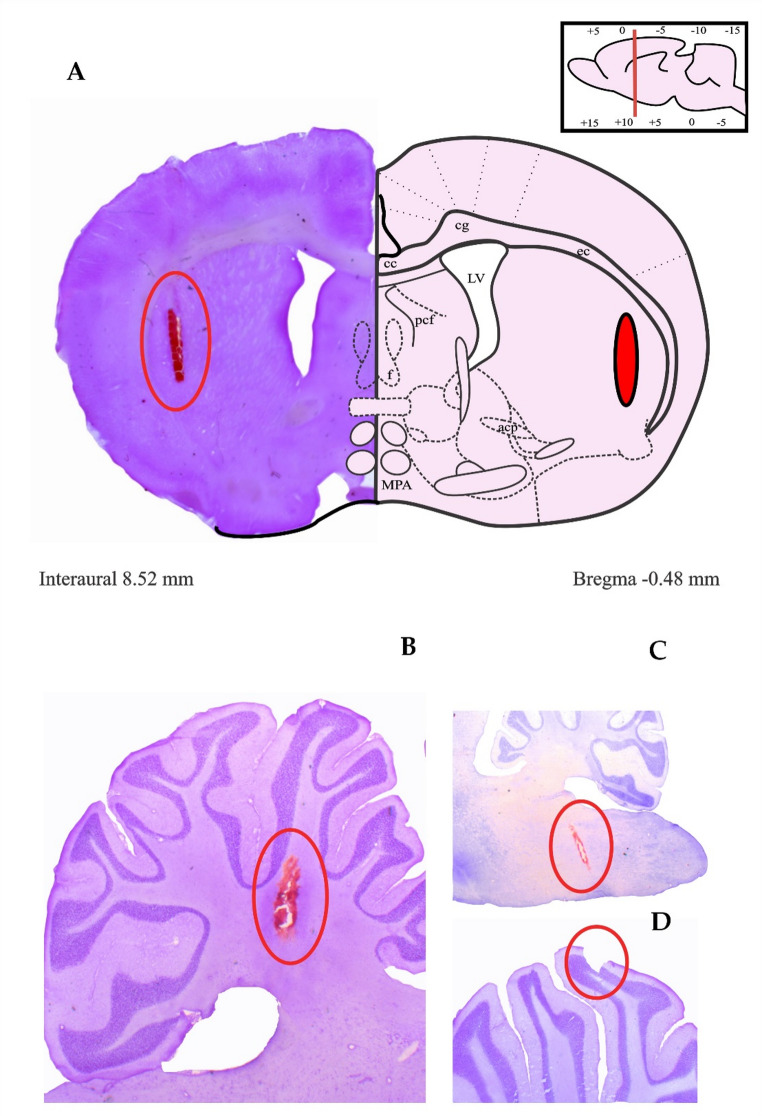
Nissl-stained brain sections. **A** Representative photomicrograph of a coronal section showing, in orange, the lesion produced by the descending trajectory of the electrode in the ventrolateral striatum (VLS). Sagittal brain sections highlighting, in orange, the recording electrode implantation sites in: **B** Dentate nucleus, **C** inferior olive, and **D** Crus II. Although no red-stained area is visible in Crus II, the presence of the implant is clearly identifiable

Following the established experimental protocol, post-mortem histological analysis was performed to validate the extent of the VLS tract lesion and the precise stereotaxic placement of the recording electrodes within the targeted cerebellar structures (IO, DN, and Crus II). Only subjects with confirmed electrode localization and lesion targeting were included in the functional data analysis. Figure 5 presents a representative example of the histological determination used to verify the lesion site and electrode tracks in the stained tissue sections.

## Discussion

The experimental model employed in this study is grounded in evidence indicating that alterations in the nigrostriatal circuit are associated with the development of Parkinsonian symptomatology [21] . Although tract lesions do not induce extensive neuronal loss, as observed in electrolytic lesions, they do interfere with the striatonigral pathway [5, 8, 9] , thereby reproducing key features of trauma-induced parkinsonism, which involves an abrupt disruption of basal ganglia circuits by an external insult, leading to structural and functional damage.

Furthermore, it is well recognized that Parkinsonian disorders do not exclusively affect the dopaminergic nigrostriatal pathway but also involve cerebellar afferent systems, including the olivocerebellar pathway [22] . In this context, the present model enables an integrated assessment of functional alterations in both striatal–nigral and cerebellar circuits, providing a framework for investigating compensatory mechanisms associated with tremor.

It is well established that the BG are directly involved in the pathophysiology of Parkinsonian symptoms (bradykinesia, rigidity, postural instability, and tremor), whereas the cerebellum is more typically associated with other motor disorders such as ataxias and coordination deficits. However, current models of motor control suggest that the cerebellum and the BG interact continuously via multiple thalamic relays, jointly influencing movement initiation, timing, and execution. In parkinsonism, compensatory mechanisms within the cerebellum may be activated [5, 11, 13, 21] . The cerebellum’s involvement in a lesion-induced parkinsonism model has provided deeper insight into how it may adapt following a VLS lesion. Although cerebellar engagement in PD and related disorders has been described previously, these findings extend our understanding of cerebellar circuits: rather than responding passively to BG dysfunction, they appear capable of actively reorganizing to compensate for or mitigate motor deficits [23] .

Therefore, the present work aimed to determine changes in cerebellar neuronal activity in an acute-lesion parkinsonism model, considering chronic alterations in structures highly implicated in the emergence of TJMs symptomatology and in baseline activity. From this perspective, our results revealed persistent differences in the basal activity of each structure following the VLS lesion. In particular, the IO, a central structure in cerebellar modulation, exhibited early changes that subsequently evolved into a progressive decline in activity within the lesion group. This pattern suggests, albeit preliminarily, a dynamic process of cerebellar adaptation. Reduced or altered olivary activity could limit, or at least modify, the cerebellum’s learning potential; indeed, this possibility is significant considering that VLS impairment may impact the IO via the communication circuit between the entopeduncular nucleus and the IO described by [24] .

Furthermore, the IO participates in motor learning, error detection, and signal timing [22, 25] . Parkinsonism might generate an overload of anomalous signals from the BG, possibly originating in the zona incerta, which reach olivocerebellar circuits. Over time, this overload could push the system toward a relatively stabilized state of active refinement in response to chronic VLS injury [24–26] . Collectively, this highlights the adaptive nature of the olivocerebellar circuit in the face of damage to interconnected motor circuits.

In contrast, the DN showed an increase in basal activity amplitude, suggesting that the cerebellum might be emitting an amplified efferent signal to counteract striatal dysfunction. This finding is consistent with other parkinsonian models reporting increased cerebellar metabolic and electrophysiological activity following BG alteration [5, 27–29] –. A sustained increase in the DN resting firing rate could represent a compensatory drive attempting to sustain motor processes normally dependent on striatal regulation [5] . Additionally, it has been demonstrated that transient suppression of Purkinje cell activity induces a rebound increase in DN activity, which could partly explain our observations [30] . Taking this into account, we propose that the progressive weekly decrease in the IO, together with the DN increase, form part of an adaptive circuit. In this model, the reduction in the olivary signal may reflect decreased transient activity transmitted via climbing fibers, which could diminish Purkinje cell activation and, consequently, reduce inhibition on the dentate nucleus, ultimately manifesting as an increase in recorded amplitudes. However, given the strong functional coupling between the inferior olive and cerebellar circuits, these changes are likely to result from the interaction of multiple mechanisms. Therefore, it remains unclear whether the observed adaptations primarily originate within cerebellar networks or arise secondarily from alterations in olivary activity. Disentangling these contributions will require future studies employing selective lesion or modulation approaches targeting the inferior olive and related pathways.

A relevant point is that, unlike the findings reported by Vásquez-Celaya et al. [5] , Crus II showed no significant differences in basal activity between groups. Even so, we observed a tendency toward reduced activity in the lesion group, which is consistent with previous reports. The lack of statistical significance could be due to the specific functional characteristics of Crus II. Although there was no *Group* × *Week* interaction, the main effect of *Group* does indicate a stable reduction in MUA amplitude, pointing to a change that is not strictly time-dependent but rather dependent on the experimental condition. The relative stability observed in Crus II under basal conditions may reflect a functional organization distinct from that of structures more directly involved in primary motor control, such as the inferior olive and the dentate nucleus. Crus II is part of the lateral cerebellar hemisphere and is closely linked to cerebro-cerebellar circuits associated with higher-order cognitive, executive, and sensorimotor functions, which are preferentially recruited during complex tasks or demanding behavioral contexts. This interpretation is consistent with studies using the same experimental model, in which Crus II neuronal activity has been shown to be affected during behaviors such as grooming [9, 31] .

Given that Crus II is strongly associated with higher-order motor and cognitive functions, including working memory, linguistic processing, and social functions, rather than basic resting motor control, its electrophysiological adaptations may be more subtle or variable under basal conditions. This may translate into minor alterations following VLS lesion during inactivity, particularly when compared with structures more directly involved in primary motor regulation [32–34] .

Furthermore, the absence of a Group × Week interaction may indicate that, in contrast to the inferior olive and the dentate nucleus, Crus II exhibits reduced time-dependent plasticity in response to VLS injury, or that its compensatory mechanisms operate through alternative pathways not directly captured by basal MUA analysis. This relative stability may represent a form of functional preservation aimed at maintaining higher-order cognitive–motor processes in the context of striatal disruption. Taken together, these findings suggest that Crus II’s involvement in lesion-induced parkinsonism is highly context-dependent, becoming more evident during active motor tasks or under conditions of increased functional demand. This issue should be further explored in future studies.

Indeed, some Parkinsonian models describe similar situations in which lateral cerebellar regions show more pronounced adaptations only under specific behavioral demands [5, 35–37] .

On the other hand, while essential tremors typically present with IO hyperactivity, the TJMs we modeled might involve distinct mechanisms [25]. The cerebello-thalamo-cortical pathway has been confirmed in tremor genesis, supporting the hypothesis that pathological synchronization of olivocerebellar neurons generates regular oscillations in cerebello-cerebral circuits [11, 38] . This anomalous synchronization could be related to the variability observed in the maximum recorded amplitude. The decrease in IO activity can be interpreted within the context of its role in motor learning. The convergence of erroneous signals from the IO with the efference copy of movement, processed via mossy and parallel fibers, constitutes an essential mechanism for inducing plasticity in Purkinje neurons [39]. Furthermore, the synchronous activation of multiple Purkinje neurons by the IO suggests that this pathway not only transmits error signals but also organizes the temporal and spatial structure of the motor sequence. This cortical synchronization is also controlled by the synchronized inhibitory discharge of climbing fibers, which is fundamental for the phase resetting of Purkinje assemblies [40].

Together, these properties help us understand how observed olivary dysfunction could alter motor coordination. Abnormalities in different types of cerebellar neurons, particularly Purkinje cells, have been associated with tremors. The repetitive architecture of the cerebellar cortex favors synchronization between the cortex and deep nuclei, a configuration that defines the oscillatory signals of tremor. Therefore, the observed changes in electrical activity could reflect alterations in synchronization between Purkinje cells and the deep nuclei, with a decrease in amplitude suggesting a reduced number or reduced efficacy of functional synapses. This type of reorganization is consistent with data indicating that the absence of Purkinje cell firing is insufficient on its own to generate tremor; rather, the phenomenon depends on a general desynchronization of the cerebellar circuit [41] . Likewise, climbing fiber hyperactivation has been associated with tremors, consistent with the idea that striatal alterations could induce a relative increase in olivary activity, generating greater climbing fiber activation and, potentially, an oscillatory state predisposing to tremor [42, 43] .

The network organization of the inferior olive, including synchronous neuronal assemblies, feedback inhibition, and phase resetting, enables the coordination of multiple motor variables and rapid corrective responses [40] , which may contribute to the observed adaptations.

In this context, the reduction in olivary signals observed in this model could reflect decreased transient activity transmitted via climbing fibers, potentially leading to reduced Purkinje cell activation and subsequent disinhibition of the dentate nucleus. Nevertheless, whether these changes represent a primary compensatory response of the cerebellum or are secondary to alterations in olivary activity remains to be determined and warrants further investigation.

The network organization of the IO, such as the synchronous activation of neuronal assemblies, feedback inhibition, and phase resetting, enables it to coordinate multiple motor-control variables and generate rapid corrections . A reduction in MUA amplitude could be due to desynchronization of these assemblies, either from the VLS lesion or from indirect effects on olivocerebellar connections [40] . Given that the BG–cerebellum circuit is bidirectional, the VLS lesion could modulate the IO via the entopeduncular–IO projection [24] . Conversely, the disynaptic projection from the DN to the striatum underscores the functional interdependence between both structures [13] . The delayed appearance of significant changes in the DN suggests compensatory mechanisms or plasticity processes. Functional connectivity of the DN with posterior cerebellar regions has been positively correlated with tremor severity, whereas its connectivity with the prefrontal cortex has been negatively associated [44], and this association could be altered in this experimental model. DN activity is modulated by Purkinje cell inhibition [30]; thus, changes in Purkinje cells following a VLS lesion could contribute to the observed patterns.

In Crus II, the decrease in MUA amplitude coincides with evidence that cerebellar cortical lesions generate disinhibition and, subsequently, deep nuclei hyperactivity [39]. Furthermore, the VLS can indirectly modify Crus II via the thalamo-cerebellar pathway [5, 31] , which involves subthalamic and pallidal nuclei that project to pontine nuclei whose mossy fibers reach Crus II. Previous studies show reduced MUA in Crus II during grooming and exploration in lesioned animals, a phenomenon attributed to striatal disinhibition [31] .

Finally, we must consider the potential influence of local tissue responses. Chronic gliosis around electrodes can alter recording impedance [45, 46] . However, the differential patterns observed, IO decreasing while DN increases, argue against a uniform artifact of inflammation, which would likely suppress activity indiscriminately. While we cannot rule out local inflammatory contributions [47–52] , the structure-specific and time-dependent nature of our data strongly supports a biological reorganization driven by the VLS injury.

A limitation of the present study is the relatively small sample size per group (*n* = 6), which may have limited statistical power to detect subtle effects. Accordingly, the absence of statistically significant differences in some parameters should not be interpreted as definitive evidence of lack of effect.

## Conclusion

The results obtained demonstrate that a tract lesion in the VLS triggers significant changes in neuronal activity within the cerebellum and IO, revealing dynamic mechanisms of neuronal adaptation associated with traumatic parkinsonism. The progressive decrease in activity in the IO and the compensatory increase in the DN suggest an active response of cerebellar circuits to mitigate motor deficits induced by striatal damage. In contrast, Crus II appeared less sensitive to changes in basal activity, likely due to its stronger involvement in complex motor or cognitive tasks. Additionally, alterations in neuronal activity during TJMs episodes confirm the differential involvement of cerebellar circuits in the regulation of specific motor symptoms. These findings underscore the need to evaluate potential local inflammatory responses caused by chronic implants, which could further clarify the global impact of invasive neural interventions on recorded neuronal activity.

## Data Availability

All data generated or analyzed during this study are available from the corresponding author upon request.
